# α-Synuclein induced toxicity in brain stem serotonin neurons mediated by an AAV vector driven by the tryptophan hydroxylase promoter

**DOI:** 10.1038/srep26285

**Published:** 2016-05-23

**Authors:** Oi Wan Wan, Eunju Shin, Bengt Mattsson, Dorian Caudal, Per Svenningsson, Anders Björklund

**Affiliations:** 1 Wallenberg Neuroscience Center, Department of Experimental Medical Sciences, Lund University, BMC A11, Lund 22184, Sweden; 2 Department of Clinical Neuroscience, Center for Molecular Medicine, Karolinska Institute, Stockholm 17176, Sweden

## Abstract

We studied the impact of α-synuclein overexpression in brainstem serotonin neurons using a novel vector construct where the expression of human wildtype α-synuclein is driven by the tryptophan hydroxylase promoter, allowing expression of α-synuclein at elevated levels, and with high selectivity, in serotonergic neurons. α-Synuclein induced degenerative changes in axons and dendrites, displaying a distorted appearance, suggesting accumulation and aggregation of α-synuclein as a result of impaired axonal transport, accompanied by a 40% loss of terminals, as assessed in the hippocampus. Tissue levels of serotonin and its major metabolite 5-HIAA remained largely unaltered, and the performance of the α-synuclein overexpressing rats in tests of spatial learning (water maze), anxiety related behavior (elevated plus maze) and depressive-like behavior (forced swim test) was not different from control, suggesting that the impact of the developing axonal pathology on serotonin neurotransmission was relatively mild. Overexpression of α-synuclein in the raphe nuclei, combined with overexpression in basal forebrain cholinergic neurons, resulted in more pronounced axonal pathology and significant impairment in the elevated plus maze. We conclude that α-synuclein pathology in serotonergic or cholinergic neurons alone is not sufficient to impair non-motor behaviors, but that it is their simultaneous involvement that determines severity of such symptoms.

Gene delivery to the brain using viral vectors has emerged as an interesting alternative to the more traditional transgenic methods for disease modeling in the central nervous system (CNS). The use of viral vectors, such as recombinant adeno-associated viruses (AAV) and lentiviruses (LV), is advantageous in that they allow expression of gene constructs in selected targets and neuronal populations, give flexibility in the timing and expression levels, and can be applied to a wide range of animal species, including rats and non-human primates. One such approach is the targeted overexpression of α-synuclein (α-syn) in midbrain dopamine neurons as a model of progressive Parkinson-like neurodegeneration driven by excess cellular levels of wild-type or mutated α-synuclein^1^,^2^,^3^,^4^,^5^. Although degeneration of midbrain dopamine neurons is at the core of the disease, and is the main cause of motor disability, it is well known that α-synuclein pathology - Lewy bodies and Lewy neurites - is more widely distributed and that other, non-dopaminergic systems are affected, including the serotonergic neurons of the brainstem raphe nuclei (RN), the noradrenergic neurons of the locus coeruleus, and the basal forebrain cholinergic neurons^6^,^7^,^8^,^9^. It is likely that α-synuclein-induced dysfunction or degenerative changes in these non-dopaminergic systems – the cholinergic and serotonergic systems in particular - contribute to the wider spectrum of symptoms, such as cognitive impairments and emotional disturbances^10^,^11^,^12^,^13^. In a recent study, Kohl and colleagues^14^ have reported reduced serotonin level and serotonin fiber density in the hippocampus, accompanied by an anxiety-like behavioral phenotype, in a BAC transgenic rat model overexpressing the full-length human α-synuclein under a pan-neuronal promoter. In these rats, however, α-synuclein overexpression was wide-spread and thus not confined to serotonergic system.

Targeted AAV-mediated expression of α-synuclein offers an interesting possibility to induce progressive degenerative changes in non-dopaminergic systems, akin to those seen in Parkinson’s disease (PD). In a pioneering study, Hall *et al*.^15^ used a α-synuclein expressing AAV2/5 vector to induce long-term degenerative changes in cholinergic neurons of the medial septum/diagonal band of Broca (MS/DB) area in rats. So far, however, this approach has not been explored in the serotonin neurons of the RN. In our initial pilot experiments we used our standard AAV2/5 and AAV2/6 vector constructs where the transgene (GFP in this case) is expressed under the control of the human synapsin-1 promoter. Although we obtained high expression of GFP in neurons outside the RN, the serotonin neurons were only poorly transduced, even at high titers of the viruses. This prompted us to generate an AAV2/6 vector where the transgene is driven by a tryptophan hydroxylase (TPH) promoter sequence developed by Benzekhroufa *et al*.^16^. This vector was highly efficient allowing us to express GFP and α-synuclein with high selectivity, and at high levels, in the serotonergic neurons of the RN.

In the experiments reported here (see Fig. 1) we have studied the impact of α-synuclein overexpression in the serotonergic neurons of the dorsal and median RN, and explored its long-term effects on serotonin-related behaviors, including spatial learning and memory, and anxiety- and depressive-like behaviors. Since the impact of impaired serotonin function is known to be amplified by dysfunction in the basal forebrain cholinergic system,^17^,^18^,^19^,^20^ we studied the effect of overexpression of α-synuclein in the presence and absence of a low dose of the cholinergic blocker scopolamine, and also in animals where α-synuclein was expressed in both the RN and the cholinergic neurons of the MS/DB area.

## Results

### Expression of the vectors

Figures 2 and 3 illustrate the efficiency of the two vector constructs, AAV-TPH-GFP and AAV- human synapsin-1(Syn1) -GFP, to transduce neurons in the RN (Fig. 2) and the MS/DB area (Fig. 3), respectively. Although the AAV-Syn1-GFP vector transduced neurons in the MS/DB with high efficiency, this vector construct was less well suited for gene delivery to the serotoninergic neurons in the RN: At the titers used here GFP was strongly expressed in neurons located outside the RN but very poorly in the serotonergic neurons in the dorsal and median RN (Supplementary Figure 1). By contrast, the expression obtained with the AAV-TPH-GFP vector, driven by the cell-specific TPH promoter, was highly specific for the serotonin neurons in the RN. Using this vector construct GFP was expressed at high levels in 50–60% of the TPH^+^ serotonin neurons (Fig. 2A–F; identified by their co-expression of TPH). In addition, GFP was efficiently expressed also in the ascending axons and axon terminals distributed throughout the di-and telencephalon (Fig. 2G–L; identified by their co-expression of the serotonin transporter, SERT).

In the MS/DB, GFP derived from the AAV-Syn1-GFP vector was expressed in large numbers of Choline Acetyltransferase (ChAT)^+^ (cholinergic) and ChAT^−^ (non-cholinergic) neurons (Fig. 3A–F). In addition, GFP was expressed at high levels in the axons running in the septo-hippocampal pathway, along the fimbria-fornix, as well as in a rich network of axon terminals throughout the hippocampal formation, bilaterally. Although we did not succeed in co-labeling the non-cholinergic neurons with the GABAergic marker glutamate decarboxylase (GAD), the expression of the vesicular GABA transporter (VGAT), in a subportion of the GFP-labeled axon terminals in the hippocampus suggests that at least part of the non-cholinergic neurons targeted by the vector were GABAergic.

### Degenerative changes induced by overexpression of α-synuclein in the RN

**Figure 1:**
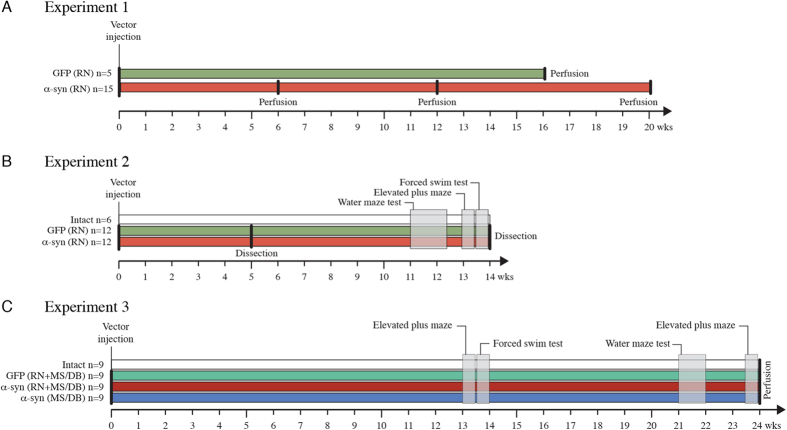
Time line and experimental design. (**A**) *Experiment 1*. Rats expressing GFP or α-synuclein in the RN were perfused for immunohistochemical analysis at 6, 12, 16, and 20 weeks after vector injection, as indicated. (**B**) *Experiment 2*. Rats expressing GFP or α-synuclein in the RN were dissected for HPLC analysis at 5 and 14 weeks post-injection. In the 14-week group the three behavioral tests - elevated plus maze, forced swim test, and water maze - were carried out during weeks 11–13. (**C**) *Experiment 3*. Four groups of rats: intact controls, rats expressing either GFP or α-synuclein in both the RN and the MS/DB area, and rats expressing α-synuclein in the MS/DB alone, were assessed for changes in performance in the three behavioral tests, starting at 13 weeks after vector injection. These rats were perfused at 24 weeks post-injection for immunohistochemical analysis.

**Figure 2:**
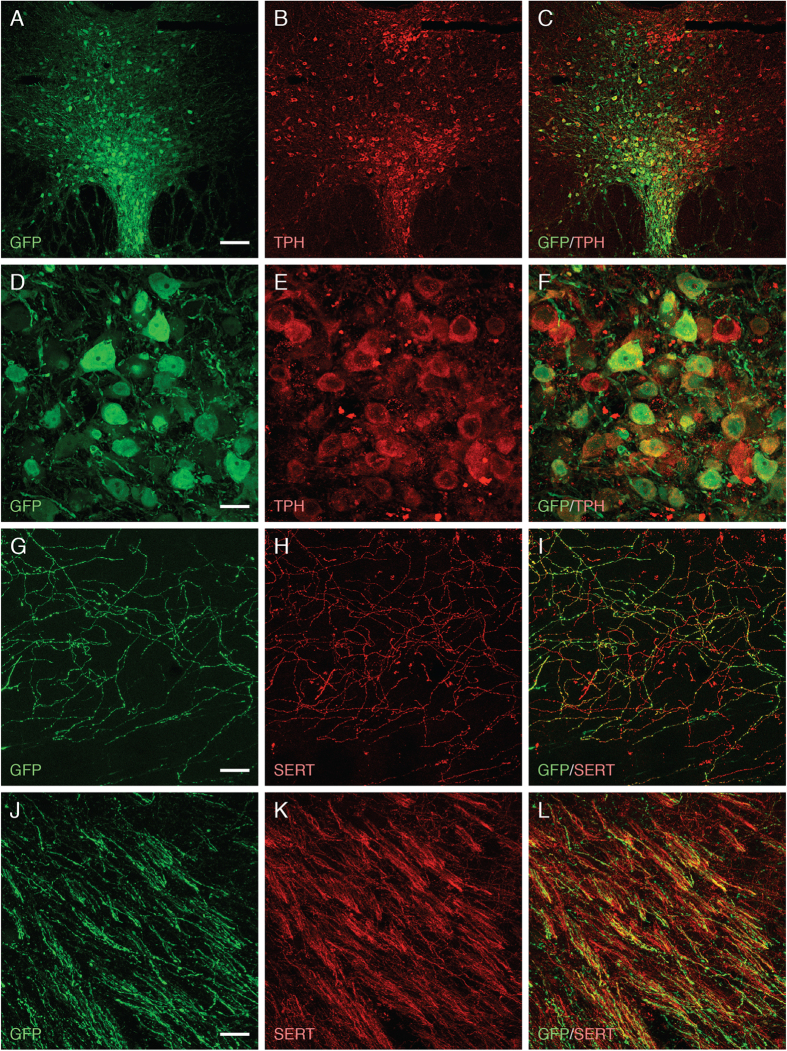
Transduction of serotonin neurons in the RN by the AAV-TPH-GFP vector. GFP, driven by the tryptophan hydroxylase (TPH) promoter, was efficiently expressed in serotonin neurons at 4 weeks after vector injection. (**A**–**F**) Double immunofluorescence staining showed co-localization of GFP (green) and TPH (red) signal in a high percentage of neurons in the DRN. (**G–L**) Co-localisation of GFP (green) and SERT (red) in serotonergic terminals in the CA3 region of the hippocampus (**G–I**) and in axons running within the medial forebrain bundle (**J–L**), showing that GFP expressed from the AAV vector was efficiently transported to fill out both axons and terminals throughout the forebrain. Scale bars: A–C: 100 μm; D-L: 20 μm.

**Figure 3:**
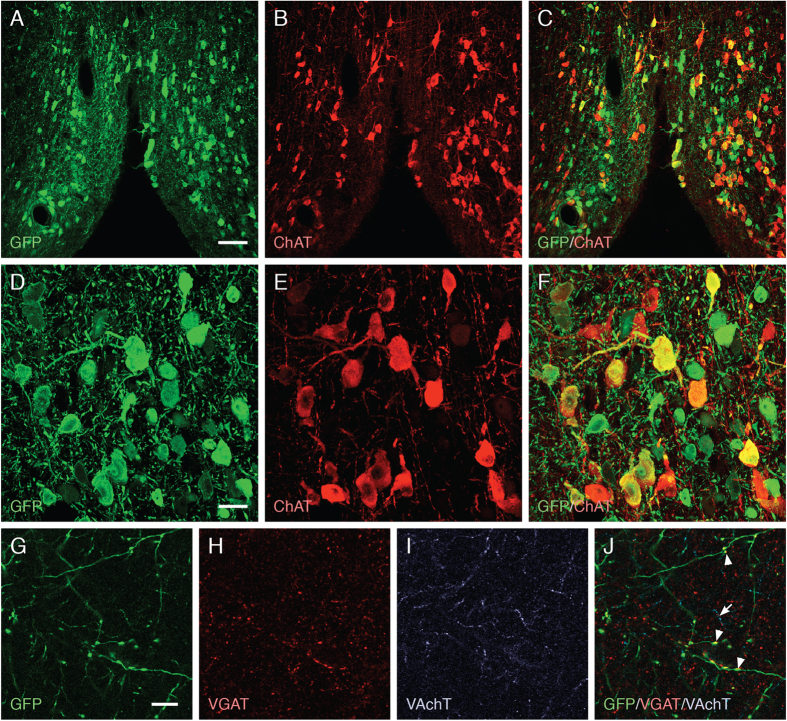
Transduction of cholinergic and GABA ergic neurons in the medial septum/diagonal band of Broca (MS/DB) area by the AAV-Syn1-GFP vector. GFP, driven by the synapsin-1 promoter, was efficiently expressed in the cholinergic neurons located in the MS/DB area at 4 weeks after vector injection. (**A–F**) Double immunofluorescence staining showed expression of GFP (green) in a high percentage of ChAT^+^ neurons (red), and also in neurons not stained for ChAT (green only). These ChAT^−^ cells most likely represent the GABA-ergic neurons present in this region (see text). (**G–J**) In hippocampus GFP was expressed in a network of axon terminals, co-localized with both the vesicular acetylcholine transporter (VAChT, arrow) and the vesicular GABA transporter (VGAT, arrowheads), confirming that both cholinergic and GABAergic neurons were transduced by the vector. Scale bars: A–C: 100 μm; D–J: 20 μm.

**Figure 4:**
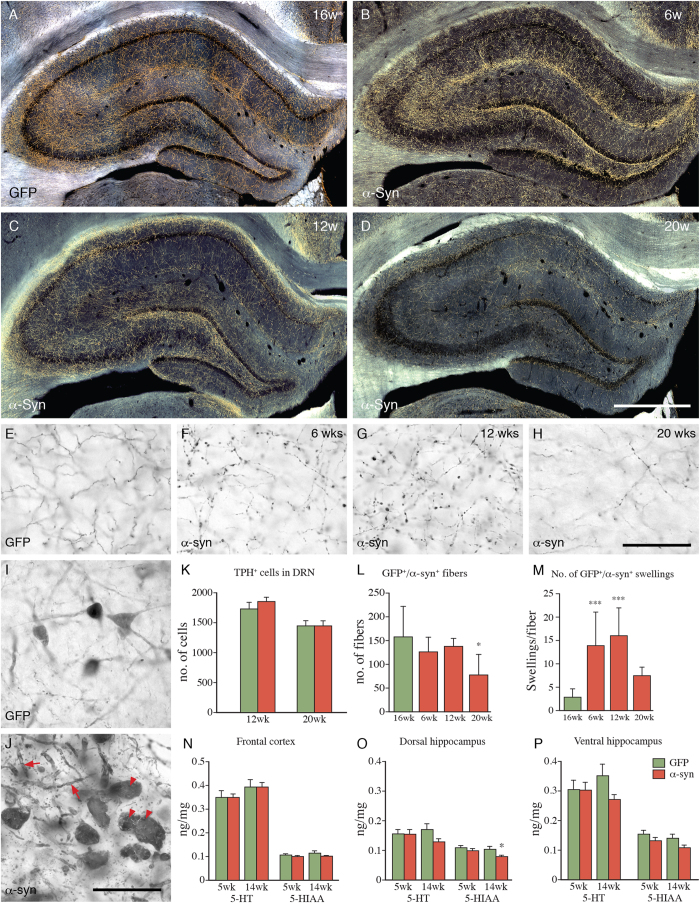
α-Synuclein expression in the RN induces axonal pathology and progressive degeneration of serotonergic terminals. (**A**) Distribution of the serotonin innervation of the dorsal hippocampus labeled by AAV-TPH-GFP injection into the RN, 16 week survival. (**B–D**) Representative cases showing progressive loss of α-synuclein positive axonal terminals in the dorsal hippocampus at 6, 12 and 20 weeks after injection of AAV-TPH-α-syn into the RN. (**E–H**) Innervation density in the CA3 area at 16 weeks after AAV-TPH-GFP injection (**E**), and the progressive loss of innervation at 6, 12 and 20 weeks after AAV-TPH-α-syn injection (**F–H**). (**I,J**) Appearance of cell bodies and neurites in the dorsal RN 12 weeks after injection of AAV-TPH-GFP (**I**) or AAV-TPH-α-syn (**J**). Note the presence of swollen and distorted α-synuclein^+^ dendrites (arrows) and the α-synuclein^+^ inclusions in (arrowheads). (**K–M**) Numbers of TPH^+^ cells in the DRN (**K**), GFP^+^ or α-synuclein^+^ fibers (**L**), and GFP^+^ or α-synuclein^+^ swellings per fiber (**M**) in hippocampus at 6, 12, 16, and 20 weeks post-injection. (**N–P**) Tissue levels of 5-HT and 5-HIAA in frontal cortex and dorsal and ventral hippocampus at 5 and 14 weeks after vector injection. Data expressed as means ± SEM (n = 6/group). Scale bars: 100 μm in B-D; 50 μm in F–J. Statistics: (**K**) Two-way ANOVA, Time: F_1,19_ = 17.02, p < 0.001; α-Synuclein: F_1,19_ = 0.56, p > 0.05; Interaction: F_1,19_ = 0.43, p > 0.05; Bonferroni’s test, p > 0.05 for GFP vs α-Synuclein at 12 wks and 20 wks. (**L**) Kruskal-Wallis test, p < 0.05, Dunn’s test, GFP 16wks vs α-Synuclein 20 wks, p < 0.05 (**M**) Kruskal-Wallis test, p < 0.001, Dunn’s test, GFP 16 wks vs α-Synuclein 6 wks, p < 0.001, GFP 16 wks vs α-Synuclein 6 wks, p < 0.001 ; (**N**) Two-way ANOVA, 5-HT, Time: F_1,20_ = 3.382, p > 0.05; α-Synuclein: F_1,20_ = 0.0004, p > 0.05; Interaction: F_1,20_ = 0.001, p > 0.05; 5-HIAA, Time: F_1,20_ = 0.6822, p > 0.05; α-Synuclein: F_1,20_ = 2.755, p > 0.05; Interaction: F_1,20_ = 0.3219, p > 0.05; (**O**) Two-way ANOVA, 5-HT, Time: F_1,20_ = 0.1345, p > 0.05; α-Synuclein: F_1,20_ = 1.910, p > 0.05, Interaction: F_1,20_ = 1.711, p > 0.05; 5-HIAA, Time: F_1,20_ = 3.068, p > 0.05; α-Synuclein: F_1,20_ = 5.661, p < 0.05; Interaction: F_1,20_ = 1.098, p > 0.05; Bonferroni’s test, GFP vs α-Synuclein: at 14 weeks, p < 0.05; (**P**) Two-way ANOVA, 5-HT, Time: F_1,20_ = 0.06868, p > 0.05; α-Synuclein: F_1,20_ = 2.101, p > 0.05; Interaction: F_1,20_ = 1.814, p > 0.05; 5-HIAA, Time: F_1,20_ = 2.744, p > 0.05; α-Synuclein: F_1,20_ = 5.971, p < 0.05; Interaction: F_1,20_ = 0.1463, p > 0.05; Bonferroni’s test, GFP vs α-Synuclein: at 5 weeks and 14 weeks, p > 0.05.

In Experiment 1 (Fig. 1A) we investigated the neurodegenerative changes induced by overexpression of human α-synuclein, using the AAV-TPH-α-syn vector, at three time points, 6, 12 and 20 weeks post-injection. The most striking changes occurred at the level of the dendrites and axon terminals. Although we did not detect any significant loss of TPH^+^ neurons in the dorsal or median RN (Fig. 4K), we observed, from 6 weeks onwards, α-synuclein^+^ cytoplasmic inclusions and the appearance of swollen and distorted α-synuclein^+^ dendritic processes (Fig. 4J), not present in the AAV-TPH-GFP treated animals (Fig. 4I). Like GFP (Fig. 4A,E), the vector-derived α-synuclein was highly expressed in the RN-derived axonal networks throughout the forebrain, allowing selective visualization of the transduced axons using the human-specific α-synuclein antibody (Fig. 4B–D,F–H). The overall innervation density, as assessed in the dorsal hippocampus, appeared unaffected at 6 and 12 weeks post-injection (Fig. 4B,C,L). At these time-points, however, the α-synuclein^+^ axon terminals had developed a striking distorted and beaded appearance (Fig. 4F,G,M). At the longest time-point, 20 weeks, the density of terminals was significantly reduced, by about 40% (Fig. 4D,H,L). Synuclein-induced axonal pathology similar to that observed in the hippocampus occurred also in other areas of the cortex, as well as in amygdala and thalamus.

Changes in tissue levels of serotonin (5-HT) and its major metabolite, 5-Hydroxyindoleacetic acid (5-HIAA), were assessed by HPLC in frontal cortex and dorsal and ventral hippocampus at 5 and 14 weeks after vector injection. As shown in Fig. 4N–P, the 5-HT and 5HIAA levels were unaffected at both time points, with the exception of a small, 20%, reduction in 5-HIAA in dorsal hippocampus seen at 14 weeks (asterisk in O).

### Changes in behavior induced by overexpression of α-synuclein in the RN

**Figure 5:**
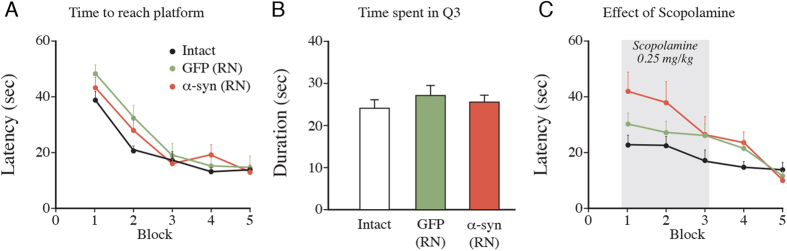
Spatial learning in the water maze in intact rats and in rats overexpressing GFP or α-synuclein in the RN, at 11-12 weeks post-injection. (**A**) Acquisition of the task was similar in all three groups. (**B**) In the final “probe test” localization of the former platform site (in quadrant 3) was similar in all three groups. (**C**) Another three groups of rats were treated with 0.25 mg/kg scopolamine, given 20 min before the first swim of the test block on day 1–3. Data are expressed as means ± SEM (n = 6 per group) Statistics; (**A**) Two-way repeated measure ANOVA, Time: F_4,96_ = 45.34, p < 0.001; α-Synuclein: F_2,24_ = 2.054, p > 0.05; Interaction: F_8,96_ = 1.133, p > 0.05 (**B**) One-way ANOVA, F_2,24_ = 0.5246, p > 0.05; (**C**) Block 1–3 has been tested with two-way repeated measure ANOVA. Time: F_2,38_ = 5.671, p < 0.01; α-Synuclein: F_2,19_ = 2.014, p > 0.05; Interaction: F_4,38_ = 1.292, p > 0.05.

In Experiment 2 (Fig. 1B) we assessed the changes in spatial learning and reference memory in the water maze (WM) at 11–12 weeks post-injection. As shown in Fig. 5A,B the acquisition of the task over the first 5 days of testing was similar in the AAV-TPH-α-syn and the AAV-TPH-GFP treated animals, and they, in turn, were not different from the intact control rats that were tested in parallel. In the second retention test, performed 1 week later, the rats were given a low dose of the cholinergic antagonist, scopolamine, administered at 20 min before the first trial each day for three days. Although not reaching a statistical significance, cholinergic blockade had a slight worsening effect only in the AAV-TPH-α-syn treated rats (Fig. 5C). This effect was observed on the first day of testing, suggesting an impaired retention of the performance learned in the previous test session. This difference gradually disappeared during the subsequent days, such that the performance of the AAV-TPH-α-syn treated rats at the end of the week was similar to that of the intact controls.

Deficits in anxiety and depression related behaviors were explored in the elevated plus maze (EPM) and the forced swim test (FST) at 13–14 weeks post-injection. The performance of the rats in the AAV-TPH-α-syn treated group did not differ from either the AAV-TPH-GFP group or the intact controls in either of the two tests (data not shown).

### Changes induced by overexpression of α-synuclein in both the RN and MS/DB

**Figure 6:**
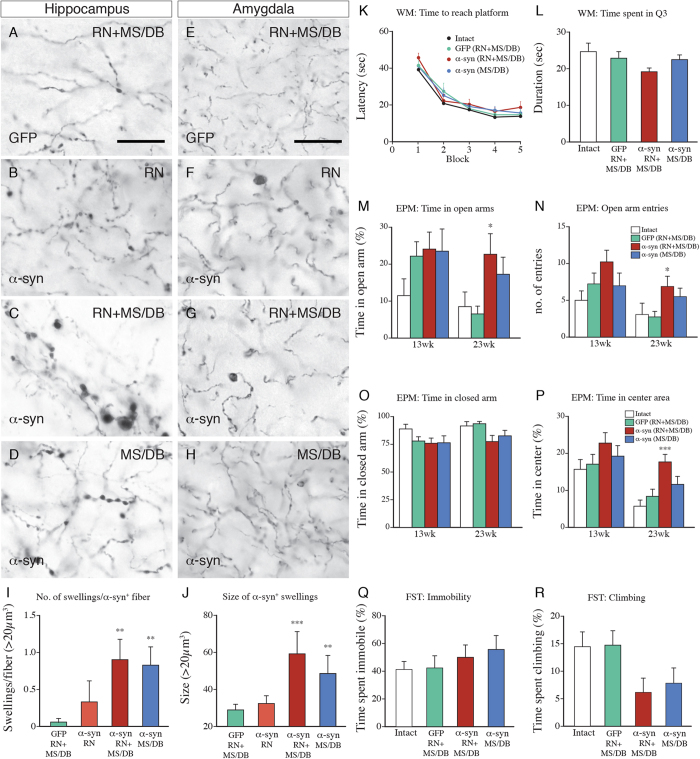
Overexpression of α-synuclein in the RN and/or MS/DB induced axonal pathology in hippocampus (**B–D**, α-synuclein stained) and amygdala (**F–H**, α-synuclein stained), not seen in the GFP-transduced controls (**A**,**E** GFP stained). Scale bar: 50 μm. (**I**,**J**) Quantification of the number of swellings per α-synuclein^+^ fiber and the size of α-synuclein^+^ axonal swellings in hippocampus of rats overexpressing GFP or α-synuclein in the RN and/or MS/DB. (**K**,**L**) Performance in the WM test showing impaired localization of the former platform site in the “probe test” in the RN + MS/DB group. (**M–P**) Anxiety-like behavior in the EPM test, performed at 13 and 23 weeks post-injection, in intact rats and in rats expressing GFP or α-synuclein. (**Q**,**R**) Depressive-like behavior was assessed in the FST, performed at 13 weeks post-injection, in intact rats and in rats overexpressing GFP or α-synuclein. Data expressed as means ± SEM (n = 9 per group). Statistics: (**I**) Kruskal-Wallis test, p < 0.001, Dunn’s multiple comparisons test, GFP vs α -Synuclein-RN + MS/DB, p < 0.01, GFP vs α-Synuclein-MS/DB, p < 0.01; (**J**) Kruskal-Wallis test, p < 0.001, Dunn’s multiple comparisons test, GFP vs α-Synuclein-RN + MS/DB, p < 0.001, GFP vs α-Synuclein-MS/DB, p < 0.01; (**K**) Two-way repeated measure ANOVA, Time: F_4,112_ = 80.56, p < 0.001; α-Synuclein: F_3,28_ = 0.6151, p > 0.05; Interaction: F_12,112_ = 0.5147, p > 0.05; (**L**) One-way ANOVA, F_3,28_ = 1.917, p > 0.05; (**M**) One-way ANOVA at 13weeks F_3,30_ = 1.497, p > 0.05; One-way ANOVA at 23 weeks F_3,31_ = 3.307, p < 0.05, Dunnett’s multiple comparison test, GFP (RN + MS/DB) vs α-Synuclein (RN + MS/DB), p < 0.05 (N) Kruskal-Wallis test at 13 weeks, p > 0.05; Kruskal-Wallis test at 23 weeks, p < 0.05, Dunn’s multiple comparison test, intact vs α-Synuclein-RN + MS/DB, p < 0.05; (**O**) One-way ANOVA at 13 weeks F_3,30_ = 1.497, p > 0.05; One-way ANOVA at 23 weeks F_3,31_ = 3.307, p < 0.05 (**P**) One-way ANOVA at 13 weeks F_3,30_ = 1.285, p > 0.05; One-way ANOVA at 23 weeks F_3,31_ = 7.894, p < 0.001; Dunnett’s multiple comparison test, intact vs α-Synuclein-RN + MS/DB, p < 0.001; (**Q**) One-way ANOVA F_3,28_ = 0.5924, p > 0.05 (**R**) One-way ANOVA F_3,28_ = 2.838, p > 0.05.

Previous studies have shown that the serotoninergic and cholinergic systems interact in the acquisition of hippocampus-dependent spatial learning and memory in the WM. Thus, the impairment seen after combined blockade of serotonergic and cholinergic neurotransmission is more profound than that induced by blockade of either system alone^17^,^18^,^19^,^20^. In Experiment 3 (Fig. 1C), therefore, we aimed to induce a combined serotonergic-cholinergic deficit by injection of AAV-α-syn in both the RN (TPH promoter driven) and the MS/DB area (synapsin-1 promoter driven). As shown in Fig. 6A–H, the overall α-synuclein-induced axonal pathology seen in these rats, as assessed in hippocampus (A-D) and amygdala (E-H), was more severe than that seen after transduction of the RN alone. Both the number (Fig. 6I) and the size of the α-synuclein^+^ axonal swellings (Fig. 6J) were 3–4-fold larger than in the rats transduced in the RN alone. Although α-synuclein-induced axonal pathology was widespread it was overall more severe in hippocampus than in amygdala or adjacent regions of the limbic cortex. We observed a 40% reduction in the number of ChAT^+^ neurons in the medial septum in the AAV-Syn1-α-syn treated animals. However, as reported earlier^15^ a similar reduction in ChAT stained neurons was observed also in the GFP-treated controls, suggesting that this was due to a reduced cellular expression of ChAT, rather than a loss of septal neurons (data not shown).

Performance in the WM test, assessed at 21 weeks after vector injection, showed similar acquisition of the task in the α-synuclein overexpressing rats (MS/DB and RN + MS/DB) and the GFP-transduced and the intact controls (Fig. 6K). In the spatial probe test, which was performed on the last (5^th^) day of testing, following removal of the platform, the RN + MS/DB rats tended to spend less time in the platform quadrant (Fig. 6L), suggesting a possible impaired retention of the memory for the location of the former platform site, not seen in the MS/DB rats or the GFP controls, or in rats with α-synuclein overexpression in the RN alone (Fig. 5B).

In the EPM test, rats in the RN + MS/DB group showed signs of reduced anxiety-like behavior. These rats tended to spend longer time in the open arms (Fig. 6M), had significantly higher open arm entries (Fig. 6N), and spent significantly longer time in the center area (Fig. 6P) compared to the intact rats, although this was not accompanied by a reduction in time spent in the closed arm (Fig. 6O). This change in performance in the EPM test was observed at 23 weeks (ANOVA in all parameters, p < 0.05), but not at 13 weeks post-injection, suggesting that it developed slowly over time. In the FST, which was performed at 13 weeks post-injection, the time spent in immobility was similar in all groups (Fog. 6Q), whereas the time spent climbing seemed to be reduced in the α-syn-RN + MS/DB group, but this change failed to reach significance (Fig. 6R, ANOVA p = 0.056).

## Discussion

Overexpression of α-synuclein in the RN induced progressive degenerative changes in the serotonin neurons that were primarily confined to the axons and dendrites. At the shorter time-points studied here, 6–12 weeks post-injection, the innervation networks seemed largely intact but the α-synuclein^+^ axon terminals had developed a striking distorted and beaded appearance, suggesting accumulation or aggregation of α-synuclein as a result of impaired axonal transport^21^,^22^. At the later time-point, 20 weeks post-injection, these changes were accompanied by an approx. 40% loss of terminals, as assessed in the CA1, CA2 and CA3 areas of the hippocampus. These changes were widespread and seen also in the amygdala and areas of the cortex. Tissue levels of serotonin and its major metabolite 5-HIAA, as determined in hippocampus and frontal cortex at 5 and 14 weeks post-injection, remained unaltered, however, suggesting that the impact of the developing axonal pathology on serotonin neurotransmission was relatively mild, at least up to the 14-week time-point.

In the three EPM and FST tests the rats in the α-synuclein overexpressing group performed at the level of the rats in the two control groups. Although lesions of the serotonin system have been shown to reduce anxiety-like behavior in the EPM test^23^,^24^, the magnitude of damage to the serotonin projections seen in the RN-α-synuclein rats was clearly not sufficient to induce any change in the performance in this test. Since only part of the serotonin neurons in the dorsal RN, about 50–60%, were transduced by our vector it seems possible that more pronounced behavioral effects could be induced if the entire serotoninergic population was affected. Interestingly, in their BAC transgenic rats with pan-neuronal overexpression of α-synuclein Kohl *et al*.^14^ observed more pronounced changes in the serotonergic system, accompanied by an anxiety-like behavioral phenotype. In these rats the hippocampal serotonin innervation was reduced by about 60%, as compared with around 40% in our AAV-transduced rats.

In the WM test it is known that lesions of the serotonin system, or blockade of serotonin transmission, has only marginal effects when administered alone, while combined serotonergic and cholinergic damage, or blockade, causes a significant impairment in both acquisition and retention of spatial memory in this task^17^,^18^,^19^,^20^. In the α-synuclein overexpressing rats we checked whether a low dose of the cholinergic blocker scopolamine might induce impairment in memory retention in the WM test. Although the RN-α-synuclein rats tended to perform worse under scopolamine (see Fig. 5C) this effect did not reach statistical significance.

Experiment 3 was designed to pursue this serotonergic-cholinergic interaction further in rats that received injections of AAV-α-syn vectors in both the RN and MS/DB. In this experiment the rats were tested at two time-points: at 13–14 weeks when axonal pathology is prominent, and at 21–23 weeks when we knew from the observations in Exp.1 that part of the serotonin innervation is lost. In the combined RN + MS/DB group significant functional changes were seen in only in the EPM test. Lesions of the serotonin system are known to induce anxiolytic behavior in the EPM test^23^,^24^, but the influence of the cholinergic system in this test is unclear. Pharmacological blockade of nicotinic and/or muscarinic receptors have been reported to be anxiolytic^25^,^26^ and an increased cholinergic tone causes anxiogenic effects^27^. To our knowledge, no previous studies have demonstrated an additive anxiolytic action of reduced serotonergic and cholinergic transmission. However, the anxiogenic effect of nicotine involves potentiation of serotonin neurotransmission^28^, supporting an important serotonin-acetylcholine interaction in the regulation of anxiety. The changes seen in the WM and FST in the combined RN + MS/DB group were overall small and did not reach significance.

Although these changes seen in the behavioral tests were subtle, and seen only in some but not all parameters measured, they support the idea that the functional impact of α-synuclein-induced damage in serotonergic and cholinergic neurons is additive. In patients with PD, α-synuclein-related pathology develops in serotonergic and cholinergic neurons in parallel with that seen in the nigral dopamine neurons, and there are data to suggest that the development of cognitive impairments and depression correlate with the extent of damage seen in these systems^10^,^11^,^12^,^13^. In line with the observations reported by Hall *et al*.^15^, using AAV-mediated overexpression of α-synuclein in the MS/DB, our findings indicate that α-synuclein- induced pathology in the RN or MS/DB alone may not be sufficient to cause changes in cognition and mood, but that it is the simultaneous involvement of these two structures, as occurs in many PD patients, that contributes to the development of non-motor symptoms. Dysfunction, or loss, of noradrenaline neurons in the locus coeruleus^8^,^24^,^29^, as well as loss of dopamine neurons in the ventral tegmental area^12^, is likely to play a role as well. Indeed, studies in the 6-hydroxydopamine lesion model of PD have shown that both motor and non-motor symptoms induced by loss of midbrain dopamine neurons are exacerbated by simultaneous damage to the cholinergic, serotonergic and/or noradrenergic systems^15^,^30^,^31^, thus adding further support to the notion that it is the combined involvement of all four systems that determine the progression and severity of symptoms in PD patients.

## Methods

### Animals and experimental design

Adult female Sprague-Dawley rats were used in all the experiment (Charles River, 225–250 g at the time of surgery) and were housed two to three per cage with ad libitum access to food and water during a 12-h light/dark cycle. The approval of the experimental protocols was made by the local research ethics committee “Malmö-Lunds djurförsöksetiska nämnd”, permit number 124–12. All the experiments were performed in accordance with the approved protocols.

The study was divided into three experiments. *Experiments 1 and 2* were designed to investigate pathological and behavior changes in rats by expressing α-syn in the dorsal and median RN. *Experiment 3* was performed to investigate the pathological and behavior changes in rats by expressing α-syn in the RN and the MS/DB area.

*Experiment 1* (Fig. 1A). Twenty rats received injections of either AAV-GFP or AAV-α-syn into the RN. The AAV-GFP injected rats were sacrificed at 16 weeks (*GFP*, n = 5) and rats injected with AAV-α-syn were sacrificed at 6, 12 and 20 weeks for immunohistochemical analysis (*α-syn*, n = 5 for each time point).

*Experiment 2* (Fig. 1B). Twenty-four rats received virus injections into the RN and killed at two time points: Half of the animals at 5 weeks, and brain tissue were dissected for HPLC analysis. The other half, including a group of 6 intact controls, was assessed for spatial learning and reference memory in the water maze (treated with or without 0.25 mg/kg scopolamine) and performance in the EPM and FST was assessed in these rats. At the end of the experiment frontal cortex and dorsal and ventral hippocampus were dissected bilaterally for HPLC analysis.

*Experiment 3* (Fig. 1C). Thirty-six rats were divided into 4 groups (n = 9/group): Intact controls; AAV-GFP or AAV-α-syn injected into the RN (TPH-driven) and the MS/DB area (Synapsin-1 driven) (*GFP-RN* + *MS/DB* and *α-syn-RN* + *MS/DB*); AAV-α-syn injected into the MS/DB alone (*α-syn-MS/DB*). All rats were assessed for depressive-like behavior in the FST and spatial learning and reference memory in WM at 13 and 21 weeks, respectively. EPM was performed in the same set of rats at 13 and 23 weeks. Rats were sacrificed at 24 weeks for immunohistochemical analysis.

### Vector Production

Production of the AAV2/6 vector was performed as described^3^. Expression of human wildtype α-synuclein and GFP were driven either by the TPH promoter^16^; *sequence listed in* Supplementary Figure 2), or the human Syn1 promoter, enhanced using a woodchuck hepatitis virus posttranscriptional regulatory element (WPRE). Genome copy (gc) titers were determined by real-time quantitative PCR. The following vector concentrations were used: AAV-TPH-GFP: 1.7 × 10^14 ^gc/mL; AAV-TPH-α-syn: 2.2 × 10^14 ^gc/mL; AAV-Syn1-GFP: 2.0 × 10^13 ^gc/mL; AAV-Syn1-α-syn: 3.9 × 10^13 ^gc/mL.

### Surgical Procedures

All surgery was performed under general anesthesia using a 20:1 mixture of fentanylcitrate (Fentanyl) and medetomidin hypochloride (Dormitor) (Apoteksbolaget), injected intraperitoneally. Virus particles were injected using a 10 μL Hamilton syringe fitted with a glass capillary (250 μm o.d.). *For the dorsal and median RN*: a total of 4 μL were distributed over 3 sites at the following coordinates (flat skull position): (1 and 2) AP: **−**6.9 mm, ML: 0 mm, DV at two depths: **−**6.4 mm (2 μL) and −8.0 mm (1 μL) below dural surface; (3) AP: **−**7.8 mm, ML: 0 mm, DV: **−**6.6 mm (1 μL). *For the MS/DB:* a total of 4 μL were distributed over 3 sites: (1 and 2) AP: **+**0.6 mm, ML: +/−0.5 mm, DV: **−**7.6 mm (1 μL at each site); (3) AP: **+**0.6 mm, ML: 0 mm, DV: **−**6.6 mm (2 μL) below dural surface. All calculated relative to bregma according to the stereotaxic atlas of Paxinos and Watson (1986). Infusion was performed at a rate of 0.2 μL/min, and the glass capillary was slowly retracted after injection. Atipamezole hydrochloride (Antisedan) and buprenorphine (Temgesic) (Apoteksbolaget) were injected to reverse sedative effects of anesthetics and to relieve pain after the surgery.

### Determination of 5-HT and 5-HIAA by HPLC

Frontal cortex (coronal cut from +2.7 mm from bregma) and dorsal and ventral hippocampus (whole hippocampus was dissected out and halved into a dorsal and ventral part) were dissected bilaterally, quickly frozen on dry ice and stored at −80 °C until further processing. Samples were homogenized in perchloric acid 0.1 N and after centrifugation at 14,000 × g for 30 minutes; 200 μL of the supernatant was filtered through a glass 0.22 μm filter (Avantec 13CP020AS). Next, 20 μL of the filtered supernatant was examined for 5-HT and 5-HIAA levels by reversed-phase HPLC with electrochemical detection. Briefly, the HPLC system consisted of a HPLC pump (LC-20AD; Shimadzu), a degasser (LC-27A; Waters), a refrigerated microsampler (SIL-20ACHT; Shimadzu), an amperometric detector (Antec Decade II; Antec), and a computerized data acquisition system (Empower; Waters). The electrochemical detector cell was equipped with a glassy carbon electrode operating at +0.7 V vs. Ag/AgCl reference electrode. Typically, 20 μL samples were injected onto a Prodigy C18 column (100 Å ~ 2 mm ID, 3 μm particle size; Phenomenex). The mobile phase 93% (wt/vol) of 94.2 mM NaH_2_PO_4_, 0.98 mM octanesulfonic acid, 0.06 mM Na_2_EDTA, adjusted to pH 3.2 with 1 M phosphoric acid and 9% acetonitrile (vol/vol). Flow-rate 0.15 mL/min.

### Tissue Processing

The rats were deeply anesthetized with 1.2 mL sodium pentobarbital *i.p.* (Apoteksbolaget) and perfused through the ascending aorta with 50 mL saline (0.9% wt/vol) at room temperature, followed by 250 mL ice-cold paraformaldehyde (4% wt/vol in 0.1 M phosphate buffered saline). The brains were removed, post-fixed overnight in 4% paraformaldehyde and cryo-protected overnight in sucrose (25% wt/vol in 0.1 M phosphate buffered saline) at 4 °C before being sectioned on a freezing microtome (Leica). Coronal sections were collected in eight series at a thickness of 30 μm.

### Immunohistochemistry

Immunostainings were performed on free-floating sections using antibodies raised against TPH (sheep, 1:500; Chemicon), GFP (chicken, 1:1,000; Abcam), human α-synuclein (mouse, 1:2,000; Santa Cruz), serotonin transporter (SERT) (mouse, 1:1,000; Chemicon), ChAT (goat, 1:500; Chemicon), vesicular acetylcholine transporter (VAChT) (Guinea Pig, 1:500; Chemicon), and VGAT (Rabbit, 1:500; Chemicon). The sections were quenched for 15 minutes in 3% H_2_O_2_/10% (vol/vol) methanol. One hour of pre-incubation with 5% (vol/vol) normal goat, horse, or donkey serum was followed by overnight incubation with the primary antibody in 5% (vol/vol) serum at room temperature. Next day, one hour incubation in 1:200 dilution of biotinylated antibody solution (Vector Laboratories) was followed by avidin-biotin-peroxidase complex solution (ABC Elite; Vector Laboratories) for another one hour. Sections were rinsed three times in potassium-phosphate buffered saline (KPBS) between each incubation period and all incubation solutions contained 0.25% Triton X-100 in KPBS. The signal was visualized using 3,3-diaminobenzidine as a chromogen, and the sections were mounted and air-dried before being dehydrated and coverslipped using DPX.

For immunofluorescence staining, similar protocol was used without the quenching step. Pre-incubation was performed with 5% (vol/vol) normal donkey serum, primary antibodies were incubated overnight at room temperature, and adequate Alexa 488, 568, and 642-conjugated secondary antibodies were incubated simultaneously. Sections were mounted and coverslipped using the FluoSave reagent (Millipore). Analysis was performed with a Leica TCS SP8 confocal microscope.

### Cell counting

Assessment of the number of TPH^+^ neurons in the dorsal RN was performed by manual counting of all cells (with visible nucleus) in every 4^th^ section through the central portion of the nucleus. The method of Abercrombie^34^ was used to correct for changes in cell size and double counting caused by cells spanning more than one section. Assessment of the total number of ChAT^+^ neurons in the MS/DB area was made by stereology according to the optical fractionator principle with the CAST Grid System (Olympus Denmark A/S), as described^3^. Every 4^th^ section covering the entire extent of the septum was included in the counting procedure. A coefficient of error of <0.10 was accepted.

### Quantification of axon terminal density and α-synuclein^+^ axonal swellings

The number of α-synuclein^+^ fibers, the number of swellings per fiber, and the size of the α-synuclein^+^ axonal swelling were determined in the CA1, CA2 and CA3 regions of the dorsal hippocampus at the level of bregma. For assessment of innervation density we adapted the “sphere counting” technique of Mouton, Goukhale and Ward^32^. Using a 100x oil-immersion objective (NA 1.35), in a brightfield microscope (Olympus AX70), a z-stack was captured throughout the thickness of the section at intervals of 1 μm, using the Velocity v 5.4.2 image analysis software (Perkin Elmer). Next, a sphere with a diameter of 19 μm (the “probe”) was created to measure fiber density within the z-stack and the points at which fibers passed through the circumference of the sphere at each z-level were counted. A score of one was given for each fiber crossing. For each probe the total number of fibers passing through the sphere was expressed as a measure of fiber density within the measured volume. For assessment of the density of α-synuclein^+^ axonal swellings a z-stack of pictures were captured throughout the thickness of the section using the same 100x oil immersion objective and images were compiled using the Velocity 5.4.2 software. This 3D reconstruction of the structure allowed the detection and counting of the swellings and dystrophic terminals. Immunoreactive structures with a volume larger than 20 μm^3^ were defined as pathological and quantified. Data are represented as the total number of axonal swellings in the three areas measured.

### Water maze test

A modified version of the water maze task originally developed by Richard Morris was used to assess spatial learning and reference memory. The maze consisted of a circular tank (180 cm in diameter), filled with room temperature water made opaque by the addition of non-toxic white paint. Extra-maze distal cues were positioned on the walls around the tank. A 15 cm-wide circular platform was fixed to the bottom of the tank and submerged 2 cm below the water surface to remain invisible to the animals. A paradigm designed to evaluate reference memory was implemented.

In the version of the test assessing reference memory, the animals were given four trials (1 block) per day over five consecutive days. Four orientation points, 90° apart, indicated as quadrant 1 (1), quadrant 2 (2), quadrant 3 (3) and quadrant 4 (4), served as starting positions, while the platform remained in a constant position (quadrant 3). On each day, the rats were released from a different position and given 60 sec to locate the platform and climb onto it. Animals were given an inter-trial time of 30 sec, during which they remained on the platform. The latency to find the platform was recorded using a computer-based video tracking system (EthoVision 3.1.13). A probe trial was performed at the end of the last day when the training, in which the platform was removed from the pool. Rats were allowed to swim for 60 seconds and the time spent in each quadrant was recorded. In Exp. 2 this training schedule was followed and scopolamine hydrobromide (Sigma) was injected *i.p.*, at a dose of 0.25 mg/kg (dissolved in saline at 2.5 mg/ml) 20 min before the first trial of the day.

### Elevated plus maze

The EPM consisted of two open arms and two closed arms opposite to each other (46 × 15 cm) and a central area (15 × 15 cm), elevated 70 cm above the floor. The closed arms were surrounded by black walls (23 cm height). Rats were placed in the center of the maze facing an open arm and allowed to explore the maze for 5 minutes in normal room lights. Several behavioral parameters were monitored by the Anymaze software (version 4.75), including time spent in open vs. closed arms, time spent in the central area, and number of entries to open vs. closed arms.

### Forced swim test

The swim test was based on a revised version of Porsolt’s protocol^33^. In the pretest, the rats were forced to swim for 15 min in glass cylinders (45 cm height, 20 cm diameter) filled with water maintained at 26 °C. The water was changed between every animal and adjusted to a height of 28–30 cm, a depth where the rats could not reach the bottom. Twenty-four hours later the rats were placed in the same glass cylinders for 6 min. The swim session was video-recorded, and the animals’ behavior was scored blindly by an observer not aware of the identity of the animals. The total duration of immobility, climbing, and activity were monitored over 6 min according to the criteria of Porsolt *et al*.^33^.

### Statistical Analysis

Statistical significance was assessed using the Graph-Pad Prism software for Mac OS X, version 6.0f (GraphPad Software, Inc.). All data are expressed as means ± SEM. Statistical significance was set at p < 0.05. When comparing more than two groups, either one-way ANOVA with Dunnett’s multiple comparison test or Kruskal-Wallis test (non-parametric) with Dunn’s multiple comparison test was used. When comparing groups on different time points, two-way ANOVA with Bonferroni’s multiple comparison test was used. When same animals were tested at different time points, two-way repeated measure ANOVA with Tukey’s multiple comparisons test was applied.

## Additional Information

**How to cite this article**: Wan, O. W. *et al*. α-Synuclein induced toxicity in brain stem serotonin neurons mediated by an AAV vector driven by the tryptophan hydroxylase promoter. *Sci. Rep.* **6**, 26285; doi: 10.1038/srep26285 (2016).

## Supplementary Material

Supplementary Information
