# Supplementary material for: α-Synuclein induced toxicity in brain stem serotonin neurons mediated by an AAV vector driven by the tryptophan hydroxylase promoter
Source: Sci Rep. 2016 May 23;6:26285. doi: 10.1038/srep26285 (PMC4876322; doi:10.1038/srep26285)
Supplement: Supplementary Information [file srep26285-s1.pdf]

α-Synuclein induced toxicity in brain stem serotonin neurons mediated by an AAV vector driven by the tryptophan hydroxylase promoter

Oi Wan Wan, Eunju Shin, Bengt Mattsson, Dorian Caudal, Per Svenningsson, Anders Björklund

Supplementary Figure 1

Representative picture of synapsin1-driven GFP expression in dorsal raphe. (A) tryptophan hydroxylase (TPH) immunostaing (B) GFP immunostaining at 4 weeks after the transduction. Scale bar = 1mm

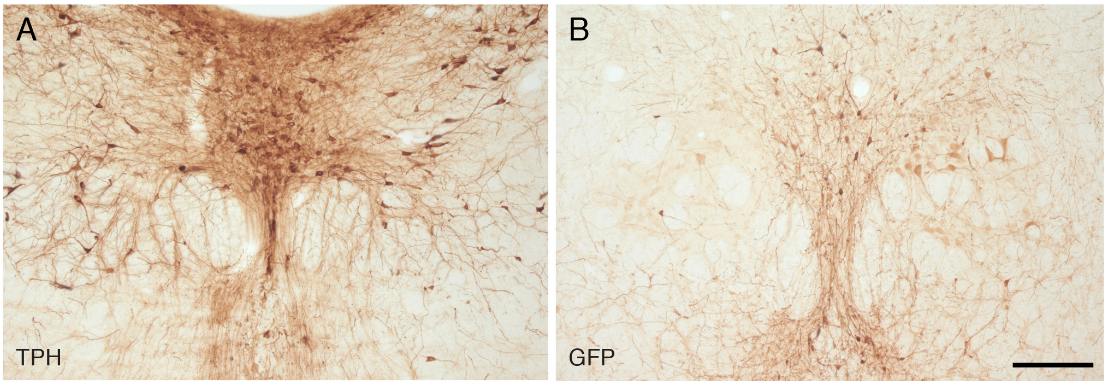

Supplementary Figure 2

Sequence of the TPH promoter used in the production of the AAV-TPH-α-syn and AAV-TPH-GFP vectors (obtained from K. Benzekhroufa et al., *Gene Therapy* 16, 681-688, 2009)

agatctggactgaccactccgtgcctttgcttttcgtgtcttctgcagagaggtcgataccctttgttctacttggttatcgcaca
acttgattctctcagccagactgtgatctccgtgggtcagtgctgcatcgaacacttctttgcatgcttgggattcagtttcagct
tactagaaactcagggctcaacaaaattttgttgagttatacatctgttctgtgtctggctctgtagtctgtcgagtctgcttttcttt
acacccttctccttctttggaagagaacacggattcccaaagtcaaattgactgaattaagatcctaccttagacattcttttgtg
ccttagtacacacacacacatacacacacacacacacacacacacagatgcacacacatacacacatatgcacacaca
catacacacagatgcacacacacacatacacatacacacagatgcacacacgcatacacatacacacagatgcacacaca
catacacatacacacagatgcacacacgcacacacatacacatacacacatgcacacgcacacataaacatacacacaga
tgcacacacgcacacacataaacatacacatgcacacacacacacacatgcacacacacatgcacatgcacacacacac
acacacaccagttttatgataactgactatgttctgacctataaacgtgcataaaaatccagaatatgattacttagcattgttag
ctattatttttacaattacttcagttaatttattaataatttctcttcctcttagcctcttagtcttcagaggaaataggagctaaatga
ttatttccagagttggatcaaataataatcctaaaccctcaagtctccagaaacagttaaaatccaaccaatgttaacatataca
gacttgttttgaagaatcttcattttgttttctatacaatccaattgtacgagtcccagattttgtttagttcttcagttaaacatgtact
cttggttaagaattttccatgtttaaaagttatttgttatttttacgtacatgagtacactgtagctgtcttcatacacaccagaaga
gggcatcaaatccccattacatatgattgtgagccacctagtggttgctgggatttgaactcaggacctctggaagagcagt
cagcactcttaaccactaagctatctctccagccccaaactttccattctttaaaggattgctaagctttcccgtggctttctaaa
gttgggaaggtacctatagaattttgtctatgcctgtcagattgctgggtctgattaagttatagatggggagcagcagaattg
catcagaaaagcatcaaagggatagtgggcctatgggcatttcatttccatatgtttcttacttagtgaagaacattctagaag
gtgagcatcttggctaagttcaagcctcgaatttcaatagcctgagtaaccttctacctggagacaccacaggttttggaaac
aggcctcttacgaaagccatcacacatacatacacataaaatttgcacatcgtaacatgtcatggtgcagaaaagccttcctt
aagctattttcttactttagaaagcacaaagcactcaaactcatagttactggaagactgagggtaaccttccaatagcaggc
cctagaattaaaattagacgactgctattttgttgcaaggaaaaaaataataataaaccctgacgcatagctctccaccatctct
tcccaaagggctactcgacctatgaaacaaacaaatctcatcatgagcacagataaccccaggcttcagacgtgtaatctga
ttgtggccatcagcaaccagaaatgagtttttctaatcagcttcccatcactcctcagtcactcatataaaggaacacaggga
ggggaggaagcgcactgctcttcagcaccagggttctggacagcgcctcgagcagccagctgccgctcaccttcctccta
catctctgccgcaccggt
